# Semantic prediction in language comprehension: evidence from brain potentials

**DOI:** 10.1080/23273798.2016.1205202

**Published:** 2016-07-11

**Authors:** Dominik Freunberger, Dietmar Roehm

**Affiliations:** ^a^ Centre for Cognitive Neuroscience, University of Salzburg, Salzburg, Austria

**Keywords:** Language comprehension, prediction, N400, P300

## Abstract

Do people predict specific word-forms during language comprehension? In an Event-Related Potential (ERP) study participants read German sentences with predictable (*The goalkeeper claims that the slick ball was easy to CATCH.*) and unpredictable (*The kids boasted that the young horse was easy to SADDLE.*) verbs. Verbs were either consistent with the expected word-form (*catch*/*saddle*) or inconsistent and therefore led to ungrammaticality (*catches/*saddles). ERPs within the N400 time-window were modulated by predictability but not by the surface-form of the verbs, suggesting that no exact word-forms were predicted. Based on our results we will argue that predictions included semantic rather than form-information. Furthermore, ungrammatical verbs led to a strong P600, probably due to task-saliency whereas correct unpredictable verbs elicited an anterior post-N400 positivity. Because the contexts were moderately constraining, this might reflect discourse revision processes rather than inhibition of a predicted word.

## Introduction

Our everyday language-use is extremely fast and efficient and a crucial contributor to this seemingly effortless capacity is prediction: Besides processing the information we receive, our brain constantly generates information to anticipate future states, actions, events, or linguistic material (e.g. Bar, 2009; Friston, 2005). Prediction is thus also known to play a central role in language comprehension (e.g. Pickering & Garrod, 2007) and new experiments on this “in fashion” research topic (Bubic, Von Cramon, & Schubotz, 2010, p. 1) seem to appear on a daily basis. This being said, some aspects of linguistic prediction remain quite vague. For instance, it is still unclear whether only meaning or also form-information of upcoming words is pre-activated. Note that with form-information we mainly mean information as to a word’s orthography during reading and information about the specific phonological realisation of a word during listening. Previous experiments that addressed this topic typically manipulated the form/meaning of target words in a variety of more or less constraining contexts. Crucially, form was mainly manipulated by using pseudowords that were or were not form-related to the target words. Pseudowords, however, do not have an entry in the mental lexicon and do not bear any meaning. Therefore, when pseudowords are used for form-manipulations, meaning is altered too. The present experiment aims to investigate whether people predict exact word-forms by using expected/unexpected forms with the same word-stem (i.e. different morphological realisations) in predictable and unpredictable contexts. This enables surface-form to be manipulated without substantially changing the meaning at the word-level.

Prediction has been demonstrated to be beneficial in a variety of cognitive domains, ranging from attention and visual processing to motor control and emotional processing (for an overview, see Bubic et al., 2010). Within the language domain, experiments have shown for example that objects that are likely to become relevant in the future are fixated upon earlier when the provided linguistic context (together with the visual environment) makes it possible to predict them (Kamide, Altmann, & Haywood, 2003) and words that can be anticipated are fixated shorter and skipped more often during natural reading (Ehrlich & Rayner, 1981; Rayner, Slattery, Drieghe, & Liversedge, 2011; Rayner & Well, 1996). Furthermore, predictable words are processed quicker (Traxler & Foss, 2000) and reanalysis is sped up in predictive contexts (Loerts, Stowe, & Schmid, 2013). Hence, words that are predictable appear to be processed more easily. To quantify semantic processing costs, researchers often use the N400 event-related potential (ERP) component. The amplitude of the N400, a negative ERP deflection, peaking around 400 Milliseconds after word-onset, is smaller when a context facilitates the retrieval of word-associated information from semantic memory (Kutas & Hillyard, 1980, 1984). It therefore has been suggested that the amplitude of the N400 can be sensitive to the degree of contextual pre-activation (Lau, Phillips, & Poeppel, 2008) and predictable words can thus lead to a smaller N400 than unpredictable words (see Kutas & Federmeier, 2011, for an overview).

When a context is restrictive and enables a word to be anticipated, linguistic features are pre-activated, that is, information is accessed and retrieved even before the word is encountered. If the prediction is correct, processing costs for this word are reduced because after reading/hearing the predicted word, no new information retrieval has to be initiated. Interestingly, when an unpredicted word is semantically related to a predicted word, the N400 is smaller compared to an unexpected word that is not related to the predicted one. For example, in a sentence where *football* is predicted (“There was nothing he enjoyed more than a good game of … ”), *baseball* elicits a smaller N400 than *monopoly*. This shows that a word’s semantic features are pre-activated and if a word shares some of these features, the processing of this word is facilitated too (Federmeier & Kutas, 1999). However, recent studies suggest that pre-activation of linguistic information is not limited to semantic features.

Results from a sign-language study can be taken as evidence that form-information is also pre-activated (Hosemann, Herrmann, Steinbach, Bornkessel-Schlesewsky, & Schlesewsky, 2013; cf. Roehm, Krebs, & Wilbur, 2012).^1^ In their study, Hosemann and colleagues found the N400 to unexpected signs to be triggered not by the onset of the sign but already by the preceding transition phase (i.e. the phase between two signs). The authors present this as evidence that “very detailed modality-specific information about the anticipated realisation of the predicted item” (p. 2234) was pre-activated. A similar conclusion came from DeLong, Urbach, and Kutas (2005; see also Delong, Urbach, Groppe, & Kutas, 2011): Here, they found a reduced N400 for indefinite articles that matched the subsequent predicted noun as compared to articles that did not match the subsequent noun (e.g. *a* versus *an* when *kite* was predicted). They concluded that their “findings unambiguously show that anticipatory processing can happen not only for conceptual or semantic features but also for specific phonological word forms” (p. 1120). However, this finding could be interpreted differently since it could be argued that the articles themselves were part of the prediction, as article-noun phrases are very common sequences and therefore might be stored as a unit in the mental lexicon and are thus pre-activated as a unit, too.

More evidence for prediction of form-information comes from Laszlo and Federmeier (2009). They ended constraining contexts (e.g. “Before lunch he has to deposit his paycheck at the … ”) with an expected word (*bank*), orthographic neighbours of the predicted word (*bark*), form-related pseudowords (*pank*), or form-related illegal letter strings (*bxnk*). In another condition, constraining contexts (e.g. “She loves the way the leaves change colour in the … ”) continued with words (*hook*), pseudowords (*jank*), or illegal letter strings (*tknt*) that were unrelated to the predicted target (*fall*). N400s were larger for all nonwords and unexpected words compared to expected words. Crucially, however, the N400 was reduced for orthographic neighbours compared to non-neighbours of the predicted word, independent of whether the word was a pseudoword, an unexpected word, or an illegal letter string. The authors concluded that specific orthographic information was pre-activated. This form-prediction influences bottom-up processing even before the lexical status of a word is processed, that is, even before the parser differentiates between words, pseudowords, or nonwords. In addition to the N400 modulation, they also observed a modulation of the late positive complex (LPC). LPC-effects were previously reported for words that violated a prediction but that were form-related to the predicted word (e.g. Vissers, Chwilla, & Kolk, 2006). This might reflect a conflict between the expected word’s form and the actually encountered form, which does not necessarily mean that the word-form per se was part of the prediction. Rather, it could be that the effect reflects the recognition of the similarity between the predicted and the encountered word-form.

There are some aspects in the study by Laszlo and Federmeier (2009) that make the results quite complex: After each sentence, participants had to indicate whether the sentence they had just read was a “normal English sentence” (p. 331) and they were told that there were no orthographical errors in the sentences. However, since the sentences contained pseudowords and nonwords, it appears difficult to disentangle the effects of orthographic relatedness from implausibility and surprise (when participants encountered a letter string such as RQCK despite being told there were no typographical errors). Furthermore, a high proportion of sentences contained pseudowords and nonwords and thus participants might have started to predict whether there would be a word or a pseudoword/nonword. Since pseudowords/nonwords were decisive for the task, this might have led to target ERP-effects that overlap with the N400 (cf. Roehm, Bornkessel-Schlesewsky, Rösler, & Schlesewsky, 2007; Sassenhagen & Bornkessel-Schlesewsky, 2015; Sassenhagen, Schlesewsky, & Bornkessel-Schlesewsky, 2014).

To sum up, increased N400 amplitudes are consistently linked to increased semantic processing costs, which occur, for instance, for unpredicted as opposed to predicted words. The semantic relatedness effects of words that were predicted/unpredicted (e.g. Federmeier & Kutas, 1999; Federmeier, McLennan, Ochoa, & Kutas, 2002; Thornhill & Van Petten, 2012) showed that meaning is pre-activated in restrictive contexts. Evidence, that form-information is also pre-activated, is, however, less coherent and might be difficult to generalise, as outlined above (e.g. DeLong et al., 2005; Hosemann et al., 2013; Laszlo & Federmeier, 2009). Although these studies do not unequivocally show that specific word-forms are predicted, they nonetheless indicate that the N400-amplitude might be sensitive to form. This assumption is further supported by studies showing that the N400 is modulated by orthographic neighbourhood size (e.g. Holcomb, Grainger, & O’Rourke, 2002), which makes it reasonable to assume that the N400 is indeed suited to test whether or not form is predicted.

It is also worth mentioning that most studies that have investigated *what* is predicted looked at relative N400 amplitude reductions: They presented unexpected items in contexts where another word was predicted, which leads to highly increased N400s. If the presented item was related (in form or meaning) to the expected one, the N400 was (or was not) relatively reduced. Thus, most of the existing evidence is based on the extent to which a prediction was violated and, notably, every form-violation was accompanied by a severe semantic violation as well (especially when pseudowords, which do not bear any meaning, were used as form-related targets). It is therefore challenging to find supportive evidence for pre-activation of form-information by inducing a word-form-prediction violation without a concurrent semantic violation.

### The current study

The current study compares ERPs to different morphological realisations of verbs that are predictable (English translation: “The goalkeeper claims that the slick ball was easy to catch/*catches”) or unpredictable (“The kids boasted that the young horse was easy to saddle/*saddles”; see Table 1 for German examples). We used second-person singular forms where an infinitive would be required, not only because this verb-form is recognisable as an inflected form of the word-stem, but also because they are distinct enough as to be clearly identifiable as a deviation (e.g. infinitival *halten* versus second-person singular *hältst*). In effect this means that the inflected verb-forms deviate with at least two letters from the correct infinitive. To ensure that participants were indeed able to differentiate between the correct and incorrect forms, they were instructed to judge each sentence’s acceptability and, crucially, before the experiment they were informed that the sentences contained semantic and grammatical deviances and that these should be judged as “not acceptable”. Furthermore, all (correct) sentences were fully plausible.

**Table 1.** Example stimuli.

COND	Example sentence	Mean CP (SD)
High-Cor	Der Torhüter behauptet, dass der rutschige Ball einfach zu fangen war. The goalkeeper claims, that the slick ball easy to catch was. “The goalkeeper claims that the slick ball was easy to catch”.	79.3% (11.4)
High-Inc	Der Torhüter behauptet, dass der rutschige Ball einfach zu *fängst war. The goalkeeper claims, that the slick ball easy to *catches was. “The goalkeeper claims that the slick ball was easy to *catches”.	
Low-Cor	Die Kinder prahlten, dass das junge Pferd einfach zu satteln war. The children boasted, that the young horse easy to saddle was. “The children boasted that the young horse was easy to saddle”.	2.5% (0.0)
Low-Inc	Die Kinder prahlten, dass das junge Pferd einfach zu *sattelst war. The children boasted, that the young horse easy to *saddles was. “The children boasted that the young horse was easy to *saddles”.	

Cor, correct; CP, cloze probability; Inc, incorrect.

Notes: Example sentence for all conditions in German, literal English, and analogous English translations, with mean cloze-values (standard deviations in brackets). Critical words are underlined.

This use of different morphological realisations (i.e. inflections) has some advantages over earlier studies, but might also have a disadvantage: On the one hand, different inflections of the same verb ensure that the meaning at the word level is relatively stable between an expected (*catch*) and an unexpected word-form (*catches*; see Table 1 for example sentences).^2^ Additionally, all critical words are real words instead of pseudowords/nonwords and this is a crucial difference in comparison to former studies, where all form-manipulations also presented a semantic expectancy violation.

On the other hand, in our study the unpredictable word-forms are inflected verbs where an infinitive is required and this renders the sentences ungrammatical. However, we do not expect the ungrammatical verbs to engender a left-anterior negativity (LAN), which is sometimes elicited by morphosyntactic violations (e.g. Friederici, Hahne, & Mecklinger, 1996), because no hierarchical and/or linking conflict of the arguments arises due to the inflected verb (cf. Bornkessel & Schlesewsky, 2006). Therefore, the ungrammatical sentences can still be unambiguously interpreted. Moreover, agreement in our materials is established between the argument of the complement phrase and the sentence-final verb (*der Ball* * … * *war*, “the ball … was”); thus no correlates of agreement failures are expected. However, one could assume that the word-form mismatch presents a *local* morphosyntactic violation within the infinitive phrase and thus might engender a LAN. Nevertheless, the ungrammaticality might influence the post-N400 ERPs: After each sentence participants had to judge the sentence’s acceptability, thus ensuring that participants did not overlook the form-manipulation. However, the acceptability judgement task also renders the ungrammatical verbs highly relevant for the task (i.e. they are even decisive for the answer). When such highly salient words are encountered, this has been shown to modulate late positivities (Sassenhagen & Bornkessel-Schlesewsky, 2015; Sassenhagen et al., 2014).

On the behavioural level, we expect grammatical sentences to be judged as acceptable and ungrammatical sentences to be judged as not acceptable. Furthermore, as it has been shown that predictability can increase task performance (e.g. Posner, Snyder, & Davidson, 1980), we thus might expect to observe a beneficial effect of predictability on reaction times (RTs) for predictable versus unpredictable sentences.

If the context enables participants to predict an *exact* word-form, we expect ERPs in the N400 time-window to be most positive for the predictable correct verbs, followed by the predictable incorrect verbs (because of the similar yet different orthography), and the most negative ERPs for the unpredictable verbs, independent of the surface-form/ grammaticality, since both deviate from the predicted word. If, however, predictions do not include exact word-form information, we do not expect ERPs within the N400 time-window to be sensitive to the word-form manipulation. Therefore, high-cloze verbs should be more positive than low-cloze verbs, independent of their grammatical status. Additionally, predictable verbs might engender a P300, reflecting the recognition of a predicted word (cf. Molinaro & Carreiras, 2010; Roehm et al., 2007). Drawing clear predictions regarding post-N400 ERP modulations is more complex. Even though all our correct sentences were plausible, we do not expect to find an anterior positivity-effect (post-N400 positivity; PNP) for the correct sentences, because the contexts are only moderately constraining in the low-CP condition (see Materials). Such PNP-effects were reported for unexpected yet plausible words in high-constraint but not low-constraint contexts (e.g. Delong et al., 2011; Federmeier, Wlotko, De Ochoa-Dewald, & Kutas, 2007; Kutas, 1993; Thornhill & Van Petten, 2012; Wicha, Moreno, & Kutas, 2004). Under the assumption that specific word-forms are predicted, another possible contributor to LPC modulations might be the conflict between predicted and encountered forms (Vissers et al., 2006).

## Methods

### Materials

In a cloze-probability (CP) pre-test, 40 German native speakers completed a total of 75 German sentences that were truncated before the critical verb. A word’s CP was then defined as the percentage used for the completion of a sentence frame. For the high-cloze condition in the EEG experiment, we selected the 30 sentences with the highest CPs and for the low-cloze condition the 30 sentences with the lowest CPs. Note that low-cloze target words were unique responses in the CP pre-test to ensure keeping the sentences plausible and, consequently, their CP was not zero. As briefly mentioned in the introduction, the context of our low-cloze condition was still moderately constraining: The mean CP of the best low-cloze candidates (which were not used as targets) in this condition was at 35.1% (SD = 13.0). For the ungrammatical conditions, we replaced the mandatory infinite target verb with a second-person singular conjugated verb-form, which presents an outright grammatical violation at this position. To quantify the difference between the correct and the deviating (i.e. infinitive versus inflected) verbs, we calculated their Damerau–Levenshtein-distance, which did not differ (*t*(29) < 1) between the predictable (*M* = 2.1, SD = .35) and unpredictable verbs (*M* = 2.1, SD = .25). We had a total of 120 experimental sentences in four conditions (high-cloze grammatical/ungrammatical; low-cloze grammatical/ungrammatical; 30 per condition). An additional 200 sentences (50% acceptable) served as filler sentences. See Table 1 for example sentences with the respective cloze-values for the four conditions.

### Participants

Twenty-three participants (18 female; mean age 22.1 years; age range 18–33 years) took part in the ERP study and were paid for the duration of the experiment. All participants were right-handed according to an adapted German version of the Edinburgh Inventory (Oldfield, 1971), had normal or corrected-to-normal vision, were native German speakers, had no known history of neurological or psychiatric disorders, and did not take part in the cloze-probability pre-test. Prior to the experiment, participants filled out a questionnaire, read the instructions, and gave written informed consent.

### Procedure

Participants read sentences from a 19-inch computer screen in a dimly lit room. Each trial began with the presentation of a fixation cross in the centre of the screen for 1500 Milliseconds (ms). The sentences were presented as single words (350 ms per word) with an inter-stimulus interval of 150 ms and the last word of each sentence was presented together with a full-stop, followed by a 150 ms blank. After each sentence participants performed an acceptability judgement task (indicated by a question mark appearing on the screen) and a probe detection task, in which they judged whether a single word that appeared on the screen was in the sentence or not (both tasks had a maximal response time of 4000 ms). In both tasks, participants responded by pressing the left or right shift-key on a computer keyboard, whereas the assignment of “yes” and “no” to the left and right buttons was counterbalanced across participants. The inter-trial interval was 800 ms. Participants were asked to avoid blinking and other movements during the presentation of the sentences. Sentences were pseudo-randomised in two versions and presented in 8 blocks of 40 sentences; the participants could take short breaks between the blocks and an experimental session lasted approximately 2.5 h overall.

### EEG recording and data processing

EEG was recorded from 25 Ag/AgCl electrodes mounted in an elastic cap (Easy Cap International, Herrsching-Breitbrunn, Germany) according to the 10/20 system (Jasper, 1958). Electrodes included: Fz, FCz, Cz, CPz, Pz, POz, F7/8, F3/4, FC5/6, FC1/2, CP5/6, CP1/2, P7/8, P3/4, O1/2. The EEG-signal was sampled at 500 Hz with a low-pass filter at 250 Hz and a software notch filter (50 Hz). Data were recorded with respect to the left mastoid reference and an AFz electrode served as the ground electrode. The horizontal electro-oculogram was recorded from electrodes at the outer canthus of each eye and the vertical electro-oculogram was recorded from electrodes placed above and beneath the left eye. Scalp impedances were kept below 5 kΩ.

Offline, all electrodes were re-referenced to the average activity of the left and right mastoids, before an ocular correction independent component analysis (ICA) was applied to correct ocular artefacts. ICA was performed in a 300 s time-window and we manually removed a maximum of two components per participant (one corresponding to the vertical EOG and one to the horizontal EOG). After the ICA-correction, the remaining EOG, movement, and technical artefacts were detected semi-automatically and removed manually. The signal was then band-pass filtered from 0.3 to 20 Hz and segmented into epochs from −300 to 1000 ms around the critical words. Trials containing artefacts or incorrect probe responses were excluded from further analyses and this resulted in an average of 93.9% for all trials. The signal was then averaged for each condition and each participant, before grand averages were computed for all participants.

### Statistical analyses

For the statistical analysis of the acceptability judgement data, mean acceptability (i.e. responses with “acceptable”) and the corresponding RTs were subjected to two separate 2 (Predictability: High, Low) by 2 (Grammaticality: Correct, Incorrect) repeated measure analyses of variance (ANOVAs) with the random factors subject and item, respectively. Note that in the item analyses predictability served as a between-factor and that for the probe detection task we only provided mean accuracies per condition.

For the analyses of the ERPs, we calculated the mean amplitudes from two time-windows (N400 time-window, 250–450 ms; late positivity time-window, 500–700 ms) for four regions of interest (ROIs): Anterior-left/right (F3/4, F7/8, FC1/2, FC5/6) and posterior-left/right. (CP1/2, CP5/6, P3/4, P7/8). The time-windows were chosen based on visual inspection. Note that the N400 time-window is identical to comparable studies (e.g. Laszlo & Federmeier, 2009).

The average amplitudes were then submitted to a 2 (Predictability: high, low) by 2 (Grammaticality: grammatical, ungrammatical) by 2 (Hemisphere: left, right) by 2 (Anteriority: anterior, posterior) ANOVA. Statistical analyses were carried out in a hierarchical manner, where only reliable interactions (*p* < .05) were resolved and *p*-values with more than one degree of freedom in the numerator were corrected according to Greenhouse and Geisser (1959). Note that effects of ROI will be reported, but not discussed unless there is an interaction with predictability and/or grammaticality. As a measure of effect size for ANOVAs, we provide generalised *Eta*-squared values (Olejnik & Algina, 2003). Only statistical results with *p* < .05 are reported. All statistical analyses were done with R (R Development Core Team, 2010).

## Results

### Behavioural data

Participants judged the grammatical sentences as acceptable and the ungrammatical sentences as not acceptable: high-correct: *M* = 97.68, SD = 15.06; high-incorrect: *M* = 1.74, SD = 13.09; low-correct: *M* = 93.90, SD = 23.96; low-incorrect: *M* = 1.74, SD = 13.10; *F*_SUBJ_ (1, 22) = 5036.06, *p* < .000, *η* ^2^ = .990; *F*_ITEM_ (1, 58) = 12661.07, *p* < .000, *η* ^2^ = .990. A reliable predictability by grammaticality interaction, *F*_SUBJ_ (1, 22) = 8.62, *p* < .01, *η* ^2^ = .039; *F*_ITEM_ (1, 58) = 5.12, *p* < .05, *η* ^2^ = .041, showed that for the correct sentences there was a significant effect of predictability, *F*_SUBJ_ (1, 22) = 10.78, *p* < .01, *η* ^2^ = .153; *F*_ITEM_ (1, 58) = 6.49, *p* < .05, *η* ^2^ = .101, which was absent in the incorrect sentences, both *F*s < 1. Resolution by predictability showed that correct sentences were judged as more acceptable than incorrect sentences regardless of whether they were predictable, *F*_SUBJ_ (1, 22) = 4662.17, *p* < .001, *η* ^2^ = .992; *F*_ITEM_ (1, 29) = 15202.39, *p* < .001, *η* ^2^ = .996, or unpredictable, *F*_SUBJ_ (1, 22) = 3571.87, *p* < .001, *η* ^2^ = .987; *F*_ITEM_ (1, 29) = 3879.94, *p* < .001, *η* ^2^ = .984.

Response times in Milliseconds for each condition were: High-Correct, *M* = 595.06, SD = 491.50; High-Incorrect, *M* = 493.77, SD = 320.63; Low-Correct, *M* = 681.10, SD = 520.55; Low-Incorrect, *M* = 505.23, SD = 364.07. The ANOVAs revealed reliable main effects of predictability, *F*_SUBJ_ (1, 22) = 12.74, *p* < .01, *η* ^2^ = .027; *F*_ITEM_ (1, 58) = 4.71, *p* < .05, *η* ^2^ = .040, and grammaticality, *F*_SUBJ_ (1, 22) = 27.07, *p* < .001, *η* ^2^ = .186; *F*_ITEM_ (1, 58) = 31.33, *p* < .001, *η* ^2^ = .229. A robust predictability by grammaticality interaction in the subject-analysis, *F*_SUBJ_ (1, 22) = 6.30, *p* < .05, *η* ^2^ = .016, revealed that – when resolved by grammaticality – RTs were shorter for predictable as opposed to unpredictable sentences only in the correct condition, correct: *F*_SUBJ_ (1, 22) = 16.12, *p* < .001, *η* ^2^ = .056; incorrect: *F*_SUBJ_ (1, 22) < 1. In addition, RTs were significantly shorter for incorrect as compared to correct sentences in both predictable and unpredictable sentences, predictable: *F*_SUBJ_ (1, 22) = 10.75, *p* < .003, *η* ^2^ = .105; *F*_ITEM_ (1, 29) = 17.94, *p* < .001, *η* ^2^ = .199; unpredictable: *F*_SUBJ_ (1, 22) = 34.11, *p* < .001, *η* ^2^ = .277; *F*_ITEM_ (1, 29) = 16.45, *p* < .001, *η* ^2^ = .260.

In the probe detection task, participants showed an almost perfect accuracy in all conditions: High-Correct, *M* = 96.38, SD = 18.70; High-Incorrect, *M* = 94.63, SD = 22.56; Low-Correct, *M* = 98.26, SD = 13.08; Low-Incorrect, *M* = 96.08, SD = 17.61.

### ERPS

**Figure 1.**
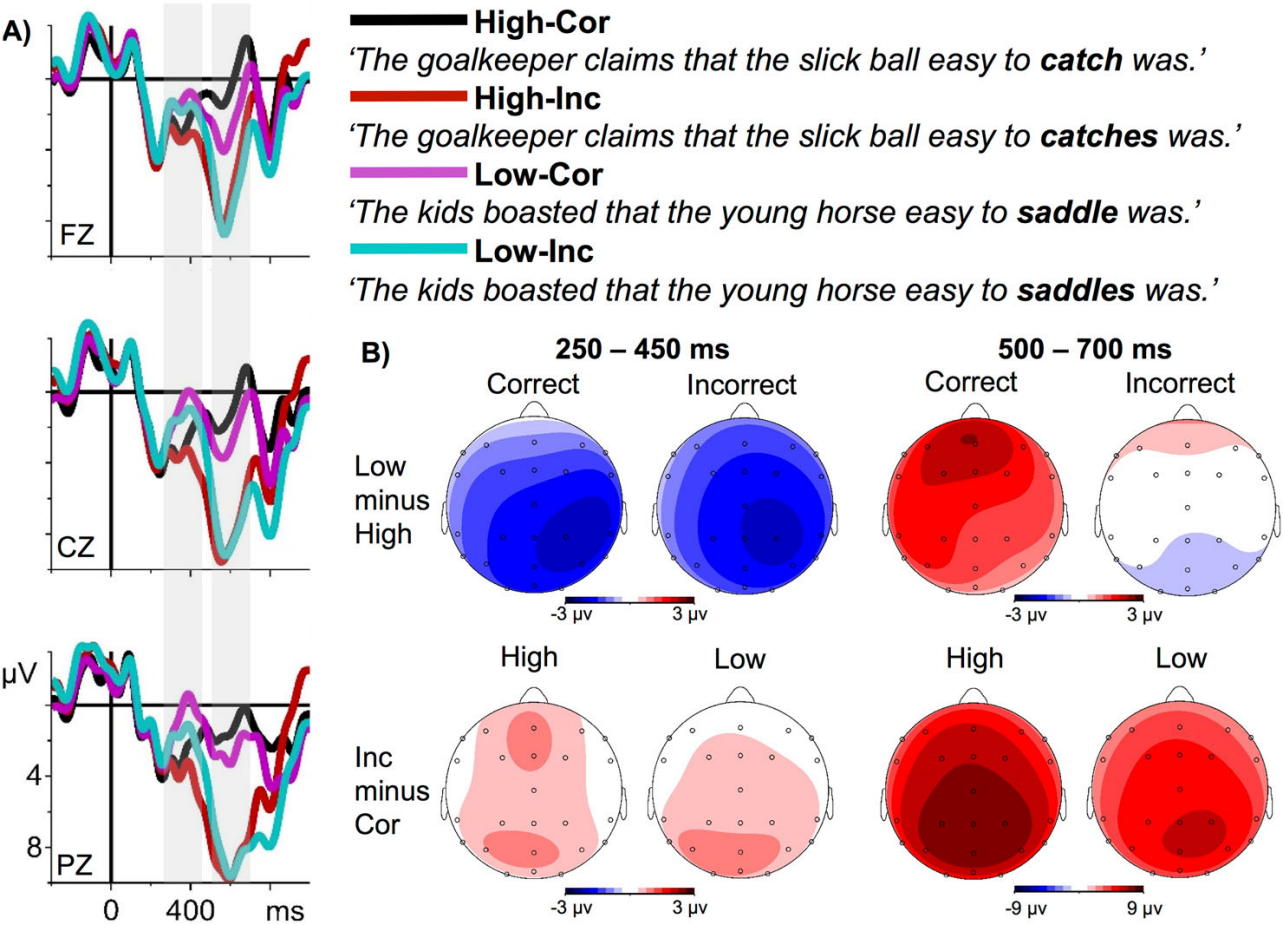
Cor = Correct, Inc = Incorrect. A. Grand average ERPs from selected midline electrodes. Time zero marks the onset of the critical words (bold in the examples given). Note that the example sentences are literal translations (for analogues translation, see Table 1). Negativity is plotted upwards. B. Differences maps from the two time-windows used for ERP-analyses.

**Figure 2.**
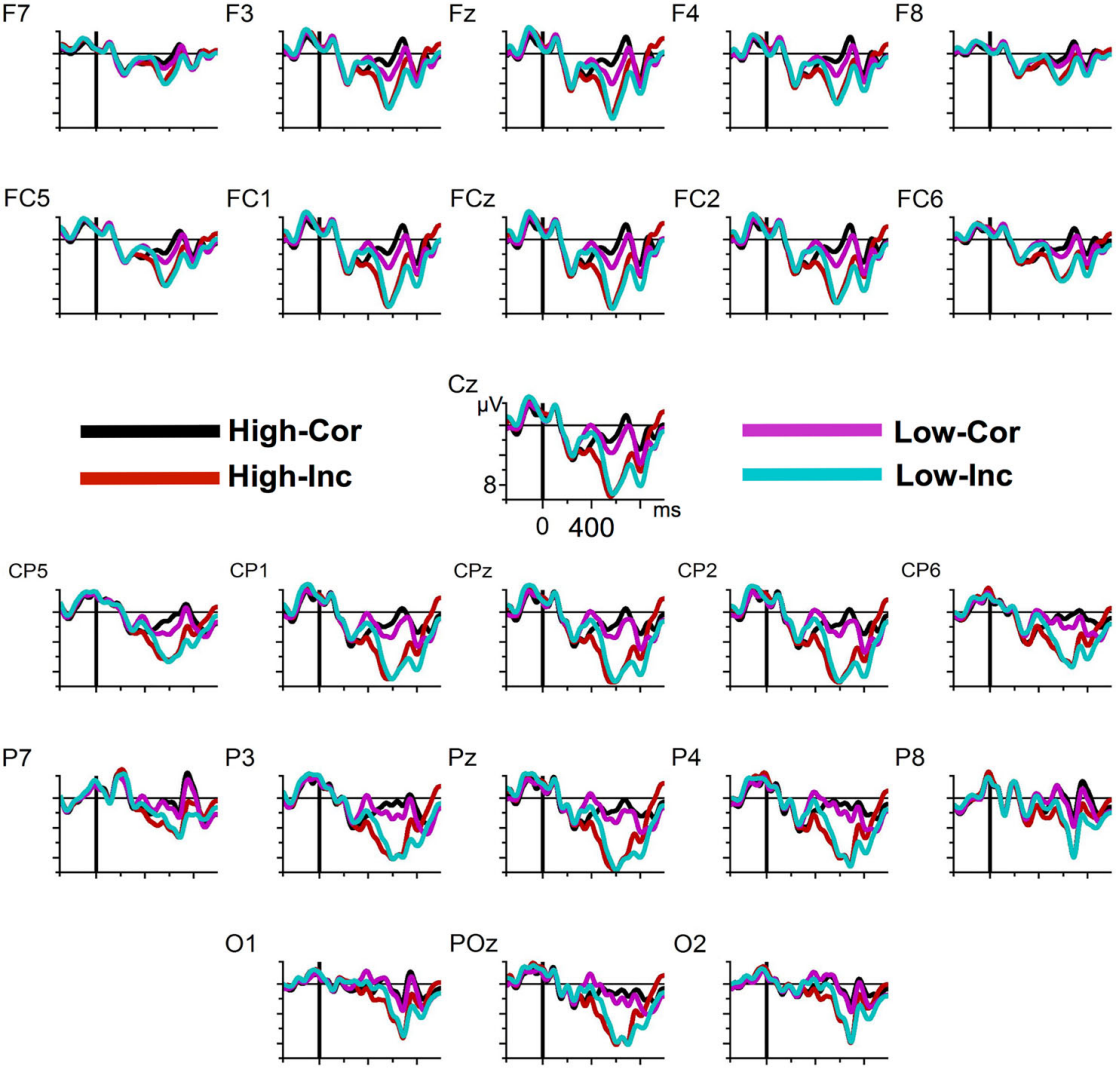
Grand average ERPs from all electrodes. Time zero corresponds to the onset of the critical words. Negativity is plotted upwards.

As can be seen in Figures 1 and 2, conditions do not differ before the evoked P2 component until about 250 ms, after which the highly predictable words, independent of grammaticality, led to a positive deflection with a peak at around 350 ms, which is clearly separable from the P2. We assume that this peak represents an instance of a P300 (cf. Roehm et al., 2007). Unpredictable verbs, on the other hand, elicited a pronounced N400 with a maximum at the central and posterior electrodes. Note that the topography of this negativity is not compatible with a LAN interpretation. From around 500 ms upwards, incorrect sentences elicited a strong positivity with a maximum at posterior electrodes. Unpredictable correct sentences led to an anteriorly distributed positivity-effect relative to the predictable correct sentences.

The ANOVA in the first time-window (250–450 ms) revealed that ERPs to predictable verbs were robustly more positive compared to unpredictable verbs, predictable minus unpredictable, *M* = 1.21, SD = 1.25, *F* (1, 22) = 46.59, *p* < .000, *η* ^2^ = .091. The effect of predictability varied as a function of hemisphere, *F* (1, 22) = 15.86, *p* < .001, *η* ^2^ = .003, as well as anteriority, *F* (1, 22) = 6.59, *p* < .02, *η* ^2^ = .002. Resolution of the interactions revealed that the effect of predictability was stronger at the right and posterior electrodes, right: *M* = 1.40, SD = 1.30; *F* (1, 22) = 51.11, *p* < .000, *η* ^2^ = .118; left: *M* = 1.02, SD = 1.18, *F* (1, 22) = 34.95, *p* < .000, *η* ^2^ = .066; posterior: *M* = 1.39, SD = 1.26; *F* (1, 22) = 47.82, *p* < .000, *η* ^2^ = .135; anterior: *M* = 1.02, SD = 1.23; *F* (1, 22) = 29.54, *p* < .000, *η* ^2^ = .058.

In the later time-window (500–700 ms), analysis revealed a reliable effect of predictability, *M* = −.55, SD = 1.67; *F* (1, 22) = 12.38, *p* < .01, *η* ^2^ = .019, and grammaticality, correct minus incorrect: *M* = −4.19, SD = 2.86; *F* (1, 22) = 78.65, *p* < .000, *η* ^2^ = .505, and a reliable interaction of predictability and grammaticality, *F* (1, 22) = 7.52, *p* < .05, *η* ^2^ = .021. Furthermore, grammaticality as well as predictability varied as a function of anteriority, grammaticality by anteriority: *F* (1, 22) = 8.73, *p* < .01, *η* ^2^ = .019; predictability by anteriority: *F* (1, 22) = 9.91, *p* < .01, *η* ^2^ = .003. The resolution of the predictability by grammaticality interaction showed that there was a reliable effect of predictability only in the correct sentences, correct: *M* =−1.18, SD = 1.60, *F* (1, 22) = 16.86, *p* < .001, *η* ^2^ = .166; incorrect: *M* = .08, SD = 1.50, *F* (1, 22) < 1. The effect of grammaticality was reliable in both, predictable and unpredictable sentences, yet stronger in the former, predictable: *M* = −4.82, SD = 2.66, *F* (1, 22) = 95.84, *p* < .000, *η* ^2^ = .661; unpredictable: *M* = −3.56, SD = 2.95, *F* (1, 22) = 41.24, *p* < .000, *η* ^2^ = .443. The interactions with anteriority indicated that the effect of predictability was significant only at anterior sites, anterior: *M* = −.84, SD = 1.66; *F* (1, 22) = 19.73, *p* < .001, *η* ^2^ = .381; posterior: *M* = −.26, SD = 1.64; *F* (1, 22) = 4.19, *p* = .053, whereas the effect of grammaticality was significant at both, but stronger at posterior sites, posterior: *M* = −4.62, SD = 2.87; *F* (1, 22) = 81.87, *p* < .000, *η* ^2^ = .555; anterior: *M* = −3.76, SD = 2.82; *F* (1, 22) = 53.20, *p* < .000, *η* ^2^ = .446.

## Discussion

### Summary of results

The current experiment investigated whether predictions that arise during sentence comprehension include form-information regarding the yet-to-be-read word. We tested the strongest case of form-prediction, that is, whether participants predict the exact surface-form of words. The critical words were either highly predictable (mean CP .79) or unpredictable yet plausible verbs (mean CP .025). The surface-form was manipulated by using a second-person singular inflection on the predictable/unpredictable verbs, where an infinitive was mandatory which consequently also rendered the sentences ungrammatical. However, as discussed in the introduction, we did not expect to find a LAN, which is sometimes elicited by morphosyntactic violations, because the inflected verb-forms do not cause hierarchical conflicts or problems in linking the arguments (Bornkessel & Schlesewsky, 2006). After each sentence participants had to judge whether or not the sentence was acceptable, which enabled us to check whether participants were aware of the surface-form manipulation. The novelty of this paradigm is that we did not use pseudowords or nonwords as form-related target words and thereby avoided outright semantic violations. We used different morphological realisations of the same verb instead, thus ensuring that the meaning remained relatively stable while varying the surface-form. We therefore hypothesised that if predictions included conceptual-semantic information but not exact word-form information, both predictable conditions should lead to equally reduced N400s.

To sum up, ERPs within the N400 time-window (250–450 ms) were not modulated by the surface-form of the target-words: There was a pronounced N400 for both (inflected and infinitive) unpredictable verbs and both (inflected and infinitive) predictable verbs led to an equally reduced N400. In addition, predictable verbs led to an early positivity (following the P2), which was also independent of the verb’s surface-form. In a later time-window (500–700 ms) we observed an interaction of predictability and grammaticality: Incorrect verbs led to a strong posterior positivity that was indistinguishable between predictable and unpredictable words. Correct unpredictable verbs, however, elicited an anterior positivity-effect relative to correct predictable verbs.

Behavioural results showed that participants were aware of the ungrammaticality in as much as incorrect sentences were judged as unacceptable and correct sentences as acceptable. Predictable sentences received marginally higher acceptability-ratings than unpredictable sentences and the faster RTs for predictable versus unpredictable sentences arguably reflect a prediction benefit (cf. Posner et al., 1980). Notably, however, this was only the case in correct sentences. Furthermore, participants might have implicitly employed an error-detection strategy, since incorrect sentences were judged faster than correct ones.

The insensitivity of the ERPs to the surface-form in the first time-window offers two interpretations of *what* was predicted: Firstly, predictions included conceptual-semantic information as well as form-information. Crucially, in this case pre-activation of form would be restricted to underspecified form-information (e.g. word-stems) because the ungrammatical input would have violated *exact* word-form predictions. Secondly, predictions included only conceptual-semantic information and no word-form information was predicted. We will discuss these two options before turning to the observed PNP-effects.

### Predicting meaning with or without form-information?

In the first time-window, ERPs were not sensitive to the grammaticality and hence the surface-form of the verbs which strongly suggests that participants’ predictions did not include exact word-form information. If participants had predicted the exact morphological realisation of the verbs, the deviating input would have led to increased retrieval costs, because the encountered information would not have not matched the pre-activated information. Consequently, the N400 elicited by the form-deviating/ungrammatical verbs would have been increased relatively when compared to the predicted verb-forms. However, Münte, Say, Clahsen, Schiltz, and Kutas (1999) showed that regular (but not irregular) verb forms can prime different realisations of the verb, suggesting that when one form is predicted, other forms of this verb are also pre-activated. In our sentences the predicted verbs were infinitive forms and the question thus arises whether the pre-activation of an infinitive verb could also include other morphological forms of that verb (e.g. via spreading activation). Infinitive forms in German have no special morphological marking and, in fact, infinitives have the same surface-form as the first- and third-person plural forms (e.g. *fangen* “to catch”; *wir/sie fangen* “we/they catch”). Therefore, when an infinitive form is predicted, it could be that personal forms are pre-activated as well. In such a case, even the incorrect verbs would have matched the prediction. Another possibility is that when a verb is predicted, this prediction includes underspecified form-information, such as word-stems. Thus, whenever a (infinitive or conjugated) verb is predicted, any form of this verb would confirm this prediction (as long as the word-stem is identical).

Indeed there are some studies suggesting that predictions that arise during sentence comprehension include specific form-information as to the yet-to-be-read word(s). For instance, Laszlo and Federmeier (2009) reported a facilitating effect for pseudowords and nonwords that were orthographic neighbours of the predicted words. Because their contexts were more restrictive than ours (CP .89 versus .79), the specificity of the prediction might have been increased to exact word-forms. Moreover, their orthographic neighbours were more similar to the expected words than in our study, which might account for the facilitation of form-related words. Nonetheless, form-related words led to greater processing costs than the predicted words (as indexed by a greater N400) despite the highly restrictive context and the high similarity between the predicted and encountered surface-forms.

The current data do not allow us to discard the possibility that (underspecified) form-information was predicted. Yet, in the light of recent results (Ito, Corley, Pickering, Martin, & Nieuwland, 2016) we suspect that in fact only conceptual-semantic information was pre-activated. Ito and colleagues ended highly constraining contexts (*The student is going to the library to borrow a* * … )* either with the predicted word (*book*), a form-related word (*hook*), a semantically related word (*page*), or an unrelated word (*sofa*). Crucially, in their experiment 1 (with a stimulus onset asynchrony, SOA, of 500 ms) they observed a facilitation of semantically related but not form-related words – as reflected in a reduced N400. In a second experiment they changed the SOA to 700 ms and found a reduced N400 for form-related targets, but only in the sentences with the highest cloze-values (CP = .94). They interpreted this finding as evidence that with an SOA of 500 ms people might not have enough time to establish predictions that also include form-information. Only in settings where participants have more time and the context restricts predictions to (almost) unique words, form-information is pre-activated too.

The setting in our experiment was highly comparable to experiment 1 in Ito et al.’s report. They also had a mean CP of .79 and an SOA of 500 ms and only observed a reduced N400 when target words were semantically related. This strongly suggests that only conceptual-semantic information was pre-activated and we thus reason also that in our experiment only meaning was predicted. The inflected (incorrect) verbs matched the predicted meaning (since they are only different morphological realisations of the same verb), as reflected in what we assume to be an instance of a P300. Roehm et al. (2007; see also Kulakova, Freunberger, & Roehm, 2014; Molinaro & Carreiras, 2010; Vespignani, Canal, Molinaro, Fonda, & Cacciari, 2009) observed such an early positivity for highly predictable words (*white*) in antonym statements (“The opposite of black is … ”). They argued that the P300 reflects the integration of a fully pre-activated word, because after word identification no new semantic information retrieval has to be initiated (as would be reflected in the N400). Similarly, Verleger (1988) suggested that the P300 reflects the closure of an active prediction (that is, adding new but predicted information), although he also linked this to task-relevancy (see also Donchin & Coles, 1988).

Notably, this association to task-relevancy can be interpreted in line with a recent account suggested by Sassenhagen et al. (2014; see also Sassenhagen & Bornkessel-Schlesewsky, 2015): They revived the debate regarding language-related late (posterior) positivities being part of a rather domain-general family of positive components, such as the P300 and P600. They interpreted this “general” positivity as a marker for the detection of salient elements, be they salient because of explicit task-relevancy, ungrammaticality, or implausibility. This is not incompatible with a view that links the P300 to predictive processing: When information is pre-activated, it certainly becomes a highly salient input, which, when encountered, elicits a P300. In the present study the P300 was not influenced by the exact surface-form of the verbs which suggests that the detection of anticipated conceptual-semantic information might suffice to elicit a P300.

Importantly, the process underlying the P300 might go beyond a bare detection mechanism: As Roehm et al. (2007) noted no lexical search is required after a predicted element is detected. Such a lexical process would be indexed by an N400. If, hypothetically, the P300 is indeed functionally similar to later positivities, the P300 might then index the integration of information on a message-level: According to a recent framework by Brouwer, Fitz, and Hoeks (2012; see also Brouwer & Hoeks, 2013), the N400 reflects semantic retrieval and post-N400 (posterior) positivities reflect the subsequent integration of the retrieved information. Since prediction means that information is retrieved before it is encountered (Huettig, 2015), no semantic retrieval is necessary after the predicted element is heard or read (cf. Roehm et al., 2007) and, consequently, no N400 is elicited. The integration of the pre-activated information into the ongoing representation might thus be reflected in the P300 (cf. Donchin & Coles, 1988; Verleger, 1988) and we reason that in our study the recognition and/or integration of the predicted conceptual-semantic information elicited such an early positivity.

### PNP

In the later time-window (500–700 ms), we observed an interaction of grammaticality and predictability: This was due to an equally strong posterior positivity in both incorrect conditions, but an anterior positivity-effect for unpredictable relative to predictable words in the correct condition. The posterior positivity for ungrammatical verbs (see differences maps 3 and 4 in the second row in Figure 1) could be explained by different factors: On the one hand, one could argue that the late positivity reflects reanalysis or reintegration due to the ungrammaticality (e.g. Kaan, Harris, Gibson, & Holcomb, 2000; Kaan & Swaab, 2003). However, the verb manipulation used here gives rise to neither conflict in interpreting the sentence (on a message-level), nor to ambiguities in argument linking (both would arguably lead to reanalysis). Hence, we do not assume that this (alone) led to the strong positivity in the ungrammatical condition.

On the other hand, as was recently argued (Sassenhagen & Bornkessel-Schlesewsky, 2015; Sassenhagen et al., 2014), late positivities might reflect a non-language-specific recognition/categorisation process, which is strongly linked to the subjective significance of the stimulus within an experiment. A stimulus’ saliency is increased by, for instance, explicit task-demands, in which the critical word becomes relevant for accomplishing the task. Also, ungrammaticality enhances stimulus saliency even when a task does not require explicit judgements (e.g. in silent reading experiments). Since the ungrammatical verbs in our experiments were decisive and thus highly significant for the acceptability judgement task, we strongly conclude that the P600 in the ungrammatical sentences is in fact due to this process.

Note that the P600-as-a-P3 account proposed by Sassenhagen et al. (2014) would not predict two subsequent positivities that arguably reflect the same function. Therefore, our interpretation that the incorrect-predictable condition led to a P300 *followed* by a P600 is not straightforwardly compatible with this view.^3^ Another possible contributor to LPC-modulations is form-deviancy: If there is a mismatch between the predicted and encountered word-form, the LPC is increased, thus indicating the detection of this mismatch. Certainly, this is strongly tied to the assumption that specific forms are predicted (e.g. Laszlo & Federmeier, 2009). However, Ito et al. (2016) demonstrated that form pre-activation is not necessary to engender LPC-modulations. The LPC might instead reflect a general detection of form-similarity independent of whether or not this form was predicted. This is in line with our findings: If the LPC would be sensitive to a mismatch of pre-activated and encountered form-information, we should observe a difference between the predictable and unpredictable condition, because the contexts were not equally constraining (the best completions in the predictable condition had a mean CP of .79 compared to .35 in the unpredictable condition). Form pre-activation should therefore be stronger in the predictable condition and, consequently, the mismatch should also be greater – as reflected in an increased positivity. As this was not the case in our data, we assume that form-prediction did not induce this late positivity. However, given the multitude of possible contributors to the observed late posterior positivity, any conclusive interpretation would be highly speculative. We can therefore only tentatively conclude that the late positivity was not modulated by word-form predictions.

An interesting and unexpected finding was the anterior positivity effect we observed for unpredictable versus predictable verbs in the correct condition (see the third difference map in the first row in Figure 1). This frontal PNP has been observed for unexpected yet plausible continuations and it arguably indicates either the detection of a disconfirmed prediction, the inhibition of the predicted word, or a message-level revision process (Delong et al., 2011; Federmeier et al., 2007; Van Petten & Luka, 2012; see also Thornhill & Van Petten, 2012). Crucially, the PNP is usually observed when the context enables a strong prediction regarding an upcoming word, that is, when the context is highly constraining. This was not the case in our unpredictable condition: The best candidates’ mean cloze was at .35, which is certainly not highly constraining. We therefore did not expect to find this PNP-effect.

Brothers, Swaab, and Traxler (2015), on the other hand, also reported a PNP in moderately and even low-constraint contexts. They argued that the PNP reflects a “post-lexical, discourse revision mechanism” (p. 146), because it was not modulated by semantic relatedness but plausibility; that is, the more implausible a continuation is, the more elaborate the revision process is (reflected in an increased PNP). The acceptability-ratings in our study might support this assumption: Correct predictable sentences were reliably judged more acceptable than correct unpredictable sentences, but although the difference was statistically reliable, it was rather small in terms of absolute numbers (a difference of 3.8%). Thus, this minor plausibility difference would have to account for the PNP-effect. Nonetheless, the Brothers et al.’s interpretation is the only one that does not call for a highly restrictive context. However, since there were major differences to our study (especially in regard to the task demands), we do not suggest a conclusive interpretation of the PNP-effect we found.

## Conclusion

In the present study we could not find evidence that exact word-forms were predicted, although the data allow for an interpretation that underspecified form-information (e.g. word-stem) was pre-activated. However, we assume that predictions included only meaning and that a match of this predicted conceptual-semantic information with the actual input elicited a P300, reflecting the recognition and possibly the integration (cf. Brouwer et al., 2012) of correctly predicted semantic information. Following the N400, we found a posterior positivity for both ungrammatical verbs, which we attribute to the detection of a highly salient/task-relevant element (Sassenhagen et al., 2014; Sassenhagen & Bornkessel-Schlesewsky, 2015). The anterior PNP-effect in the correct sentences might be due to a (partial) revision of a message-level representation that was built up during prediction (cf. Brothers et al., 2015), although this interpretation remains tentative.

In line with previous findings we conclude that in a similar experimental environment (i.e. moderately high predictability of .79 and a standard presentation-rate of 500 ms), comprehenders pre-activate semantic but not form-information. Ito et al. (2016) presented strong evidence that this assumption is compatible with models that assume that the language production system is crucially involved in generating predictions in online language processing (Pickering & Garrod, 2007). In such models, the pre-activation of meaning precedes the pre-activation of form. Form, however, is arguably only predicted in highly constraining contexts with slower input rates.

## Notes

1. Note that with “form-prediction” we refer – in line with other authors (e.g. Hosemann et al., 2013; Laszlo & Federmeier, 2009) – to the pre-activation of fairly specific features of the yet-to-be-seen/heard word. That is, in auditory experiments, listeners might predict information as to the specific phonological realisation of predictable words. In reading, pre-activation might include information as to the orthography and even visual features (see, for instance, Balota, Pollatsek, & Rayner, 1985, for evidence from natural reading suggesting that visual features are pre-activated).

2. Although fine-grained semantics between infinitive forms and inflected forms might differ (inflected verbs, for instance, contain information regarding their arguments, voice, temporal information, person, and number), different morphological realisations of the same verb share a “core” meaning that is decoded in the word-stem.

3. Yet, it could be that both positivities reflect the same mechanism (i.e. detection of relevant/salient input) and only the source of this relevancy is distinct for the two positivities (i.e. prediction for the P300, task-relevancy for the P600). Consequently, the positivities are cascaded. This, however, is highly speculative and requires further research.
